# Counting more than beans: A different take on key performance indicators for core facilities

**DOI:** 10.1038/s44319-026-00762-z

**Published:** 2026-04-08

**Authors:** Farzaneh Ghazavi, Alexander Botzki, Stefaan Derveaux, Christine Durinx, Gert Van Isterdael, Geert Van Minnebruggen, Saskia Lippens

**Affiliations:** 1 VIB Technologies, VIB, Flanders Institute for Biotechnology, Ghent, Belgium; 2 VIB, Flanders Institute for Biotechnology, Ghent, Belgium

**Keywords:** Economics, Law & Politics, Methods & Resources

## Abstract

Core facilities are central to life-science research, yet performance frameworks borrowed from academia or administrative settings often miss what matters most in a technology-driven, service-focused environment. This article proposes a practical approach to selecting strategic KPIs to steer priorities and guide decisions.

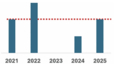

Core facilities have become indispensable for research in the life sciences as they provide the research community with access to cutting-edge, often expensive, technology and high-quality services. Institutes that have invested in centralized research infrastructure profit from economies of scale and from being at the forefront of scientific innovation (Meder et al, 2016). Yet, the concept of core facilities is relatively new in the history of scientific research. How institutes and their facility managers should evaluate performance in terms of supporting breakthrough science is, therefore, an ongoing discussion.

Since core facilities manage centralized infrastructures that serve a large number of users, an evaluation of their performance is relevant for multiple stakeholders, not just the host institution. There is a financial imperative to ensure that expensive technology is well-managed, accessible to the scientific community and supported by a sustainable model that covers ongoing maintenance costs. There is a scientific imperative to make sure that the research community benefits from the core facility’s technologies and services in terms of research output and quality. Performance measures that demonstrate these goals therefore support the facility’s continuous operation and ability to offer a portfolio of state-of-the-art services. Moreover, regular reviews of performance against a set of well-defined measures help the facility’s and host institution’s management with strategic planning and investments.

Regular reviews of performance against a set of well-defined measures help the facility’s and host institution’s management with strategic planning and investments

However, traditional output metrics borrowed from academia or administrative settings often fail to fully capture the extent of innovation and impact provided by a technology-driven, user-centered service. This results in a significant disconnect between what is measured and what is the real value of core facility operations for its stakeholders. In this paper, we therefore propose a structured and practical approach to assess core facility performance, based on efficacy, efficiency and proactive innovation.

## Metrics vs KPIs

To a certain extent, the results and activities of core facilities can be described with metrics. However, it’s essential not to confuse metrics with indicators or with their more specific subset: key performance indicators (KPIs). **Metrics** are measurable values: raw counts or calculated rates. **Indicators** are selected metrics used to monitor performance and operational status and provide insight into what is happening. **KPIs** are a small set of critical indicators linked to strategic objectives that are used to steer priorities and track progress against targets (Clissold and Itano, 2025). Both metrics and KPIs are essential for effective performance management. While metrics provide broad operational visibility, KPIs focus on the measures that matter in terms of assessing whether a facility achieves key goals.

For example, user satisfaction scores from post-service surveys are commonly tracked as a metric. Interpreted over time, they also serve as an indicator of perceived service quality—whether, for instance, user satisfaction is improving or declining. When this measure is selected as a strategic priority and tracked against a defined target, such as “maintain an average user satisfaction score of more than 4.5 out of 5”, it functions as a KPI, linked to the strategic objective of delivering high-quality service.

Interpreting metrics is not merely a mathematical exercise. A first issue is that single-point snapshots should be avoided; instead, tracking metrics over time to observe trends or patterns, is much more valuable. However, even then, there is a risk in drawing conclusions purely on data. Without the proper context, simple metrics are often insufficient to evaluate whether a core is performing well or not. As with any performance evaluation in a highly specialized field, core facility performance and KPI evaluation are complex matters that depend on various factors, including the kind of technology that is offered.

Without the proper context, simple metrics are often insufficient to evaluate whether a core is performing well or not.

Even though monitoring core facility KPIs requires significant effort, it is an essential practice that offers numerous benefits. Embracing KPIs as valuable tools rather than administrative burdens can lead to greater success and sustainability in the constantly changing landscape of scientific research. KPIs provide a clear picture of a facility’s performance and serve as strategic indicators, guiding a facility towards achieving its goals and maximizing its impact. Moreover, robust evaluation of KPIs is crucial for infrastructure grant submissions. Reviewers look for evidence of a facility’s ability to deliver the right support to contribute to scientific advancement, and KPIs can provide this evidence in a clear and quantifiable manner.

## Three main domains

The foremost measure of a core facility’s success is its alignment with the overarching mission of the institution, typically to support scientific research and innovation through providing access to high-quality technologies and expertise. This mission differs from the expectations for scientists in research labs, who need to produce relevant and high-quality research with adherence to ethical standards—transparency, reproducibility of results, good scientific practice and so on—train students, secure funding, set up scientific collaborations and inform the lay public. Core facilities do, of course, contribute to these outcomes, but in an indirect manner.

The first task, then, is to define the areas in which a core facility has to excel to determine whether it is functioning well. We see three main questions that need to be addressed: Is a core facility **effective** in delivering scientific value, supporting research outputs, enabling users and driving innovation? Is the core facility functioning **efficiently and reliably**? Is a core facility **proactively** taking measures to remain state-of-the-art and future-proof? These domains serve as the foundation for defining high-level KPIs in core facility management (Tables 1–3) (Turpen et al, 2016).

**Table 1:** Effectiveness of a core facility.

Domain	Related metrics (examples)
**Scientific contribution**	# co-authorships, # publications acknowledging the core, # citations, # datasets generated
**Community reach and engagement**	# unique users, # unique labs/teams, # projects supported, user base composition
**Training & user enablement**	# users trained/certified, # workshops, # hands-on trainings, training satisfaction, % trainees passing competency check

**Table 2:** Efficiency, reliability, and quality.

Domain	Related metrics (examples)
**Operational performance (speed, availability, and capacity)**	Turnaround time, uptime/downtime, queue time, instrument utilization (% booked vs available), throughput (# runs/samples delivered), staff time allocation
**Quality & compliance**	Success rate, error/rework rate %, QC pass rate %, #deviations, SOPs up to date (%), training completion (%)
**User experience**	Satisfaction score, time to first response, complaint rate
**Financial sustainability**	Cost recovery rate %, net balance, subsidy level, cost per run

**Table 3:** Future-readiness and innovation.

Domain	Related metrics (examples)
**Technology scouting and adoption**	# tech scouting activities (events, vendor demos), # pilots started, % staff trained on new tech, # users adopting new services
**Portfolio evolution**	# new services added, # services retired, stage-gate progression (pilot→validate→routine), % capacity protected for innovation, time-to-routine for new workflows, # major method upgrades
**Innovation output**	# workflow improvements, # new methods validated, # in-house tool/workflow development, # automation/digitalization improvements, #“flagship” workflows/platforms established, # feasibility studies enabling breakthroughs, # patents filed
**Collaboration and knowledge sharing**	# internal cross-core collaborations, # external partnerships, # joint grants supported/submitted, #outreach activities, # benchmarking participation

Only the core-specific subset of these metrics is defined as KPIs: those that are most important for achieving the strategic goals, with a clear target, an accountable owner, a regular review schedule and an agreed action plan when performance falls below the target.

## Measuring scientific value and community enablement

As core facilities should enable and support the scientific output of their users, it is relevant to demonstrate a facility’s contribution to research using metrics such as the number of publications supported, scientific authorships, acknowledgements and citations of method papers. In general, a higher value indicates a stronger contribution to scientific output (Kivinen et al, 2022). Scientific authorship, however, should be interpreted with caution: authorship practices vary across fields and teams, and core facility contributions are not always reflected in the author list or acknowledgements. Beyond publications, a facility’s role in advancing science can also be reflected in innovation-related outcomes. Supporting metrics may include patents filed, citations on major discoveries, and the development and implementation of new methods and workflows.

Usage metrics, such as the number of projects supported, and the number of individual research labs and scientists served, provide indirect evidence of the facility’s scientific relevance and reach and can therefore be used as indicators of community engagement. While detailed demand measures are primarily used for operational management and capacity planning, consistently high demand over time can also be interpreted as evidence of scientific value, as it reflects that the research community continues to rely on the facility.

Another form of supporting the scientific community is through educating and training. Related metrics, such as the number of trained users, workshops or seminars, reflect the facility’s engagement to empower the user community with know-how and quality standards in specific technology fields and can be used as indicators of user enablement.

Finally, these effectiveness-related measures should be interpreted in context, as no single metric captures the full contribution of a core facility. Co-authorship in particular is not fully under the control of the facility and may underrepresent actual contributions. This is also evident in training metrics: some technologies require extensive user training, whereas other services have naturally a very low or no demand for user training. A more balanced assessment, therefore, uses a combination of indicators to cover scientific outputs, innovation deliverables, community reach and training to capture the facility’s contribution more reliably (Table 1).

## Optimizing core facility operations

A facility can be managed in many different ways, and there is no blueprint of which scenario is best, not even for facilities in the same technology domain, because local context will always have an influence on the optimal approach. Innovations and improvements in a facility don’t just happen at the technical or scientific level but also include advances in how the facility is run, its management, processes and workflows. Users expect and deserve access to technology as efficiently as possible, but not at the expense of scientific quality. Therefore, performance management in core facilities often focuses on how quickly and consistently services are delivered, alongside service quality, which can be formalized as KPIs where relevant (Moore and Roederer, 2009; Gregory, 2020) (Table 2).

Selected metrics should help identify opportunities to improve process efficiency, service continuity and productivity

Examples of operational metrics include time between a request and result delivery (turnaround time), equipment availability and downtime, and resource allocation. Operational demand and capacity needs can be quantified through metrics such as instrument usage, the number of samples processed and service throughput. These metrics support efficiency-related decisions, for example, by indicating whether capacity is sufficient or whether equipment replacement or expansion is needed. Service quality can be monitored using metrics such as experiment success and error rates, adherence to standard operating procedures (SOPs), and compliance with safety and regulatory standards.

Efficient financial management is an important dimension of performance, reflecting whether a core facility is financially future-proof and sustainable (O’Toole and Marrison, 2024). A common metric is financial balance between operating costs and revenues; however, this only shows profitability, which in certain technology domains can be very challenging to achieve without compromising fair and affordable access for the user community. Therefore, it is often more informative to track cost recovery, that is, the proportion of operational costs covered by user fees. This metric is not informative on its own but should be tracked over time and compared to similar facilities with regard to services offered and subsidies available, to help understand whether a facility can continue to afford its operations. Other useful financial metrics to consider include diversity of revenue streams and participation in instrument grants.

## Proactive strategy: innovation in core facilities

In the world of core facilities, where technology plays a central role, continuous innovation is imperative to make sure that the facility is able to offer state-of-the-art services. It is essential that a core is able to shift gears when the next disruptive application becomes crucial for research. A core must therefore actively anticipate future scenarios and prepare for the next emerging technology or workflow, both by adopting innovations from the broader scientific and technological landscape and by fostering internal innovation.

The frequency of scouting and testing new technologies, along with training staff in new techniques are metrics that can serve as indicators of whether a core is successfully adopting new methods and maintaining adequately trained staff to support new technology. But staying up to date with all kinds of new technology is not trivial and requires a proactive approach. Outreach and networking are important to stay informed about current developments and emerging trends in the community.

Collaboration takes this a step further. Core facilities should be involved or sometimes take the lead in driving technological innovation. This can be evidenced through metrics or indicators such as participating in grant applications with PIs, and the establishment of partnerships with vendors, engineering teams or other external stakeholders.

As such, facilities play a unique role in the life-science tech ecosystem. Expert staff may see opportunities to improve the ongoing service or think outside the box and develop completely new ideas. The number of technology or workflow improvements, in-house developments and co-developed innovations can serve as indicators of an innovative culture. Additionally, one can focus on the number of new services launched, the number of automation/digitalization improvements, user adoption of innovations, and external funding or recognition received for innovative activities. A selected subset of these indicators may be used as KPIs to evaluate how actively and effectively a facility contributes to technological advancement (Table 3).

Because the mission of cores is to support researchers, the user community is in a privileged position to provide feedback, which can be gathered in various ways. Typically, core facilities conduct anonymous user satisfaction surveys, but feedback can also come from face-to-face discussions with a “critical colleague”, or structured approaches such as meetings with a user committee, focus group or advisory board. Such feedback serves as a direct measure of user satisfaction and service effectiveness. The purpose is not to strive for a perfect score but to identify areas for improvement and unique strengths of the core facility. These insights help to guide future strategic decisions and directions, and foster open communication and building relationships. Asking the right questions is key to this process. While survey outcomes provide valuable metrics, selecting a small set of them as strategic priorities and tracking them against defined targets—such as satisfaction scores, Net Promoter Scores or response rates—allows them to work as meaningful KPIs.

## Ongoing effort

It is clear that the evaluation of core facility performance using KPIs is a multifaceted task that requires a high level of expertise, contextual understanding, and a nuanced approach to different technologies (Box 1). It includes a structured methodology and the right tools to navigate effectively. The process is far from straightforward, as it involves analysis of multiple metrics to arrive at a comprehensive reflection of the facility’s performance (Kos-Braun and Gerlach, 2022). Efforts to foster community discussion and practical implementation of KPIs are ongoing, including dedicated workshops aimed at engaging the broader scientific community.

It is clear that the evaluation of core facility performance using KPIs is a multifaceted task that requires a high level of expertise, contextual understanding, and a nuanced approach…

In a fast-evolving environment, a facility has to respond to new challenges and opportunities to remain relevant to the scientific community. There is a continuous process of decision-making for day-to-day management and for implementing changes to keep pace with all these influences on the operations of a core. Longitudinal tracking of metrics and KPIs gives a dynamic view on the activities of a core, which provides valuable insights into whether a facility is geared towards enabling scientific research and remains adaptable to future needs.

Box 1 Example KPI dashboard: interpreting trends in context

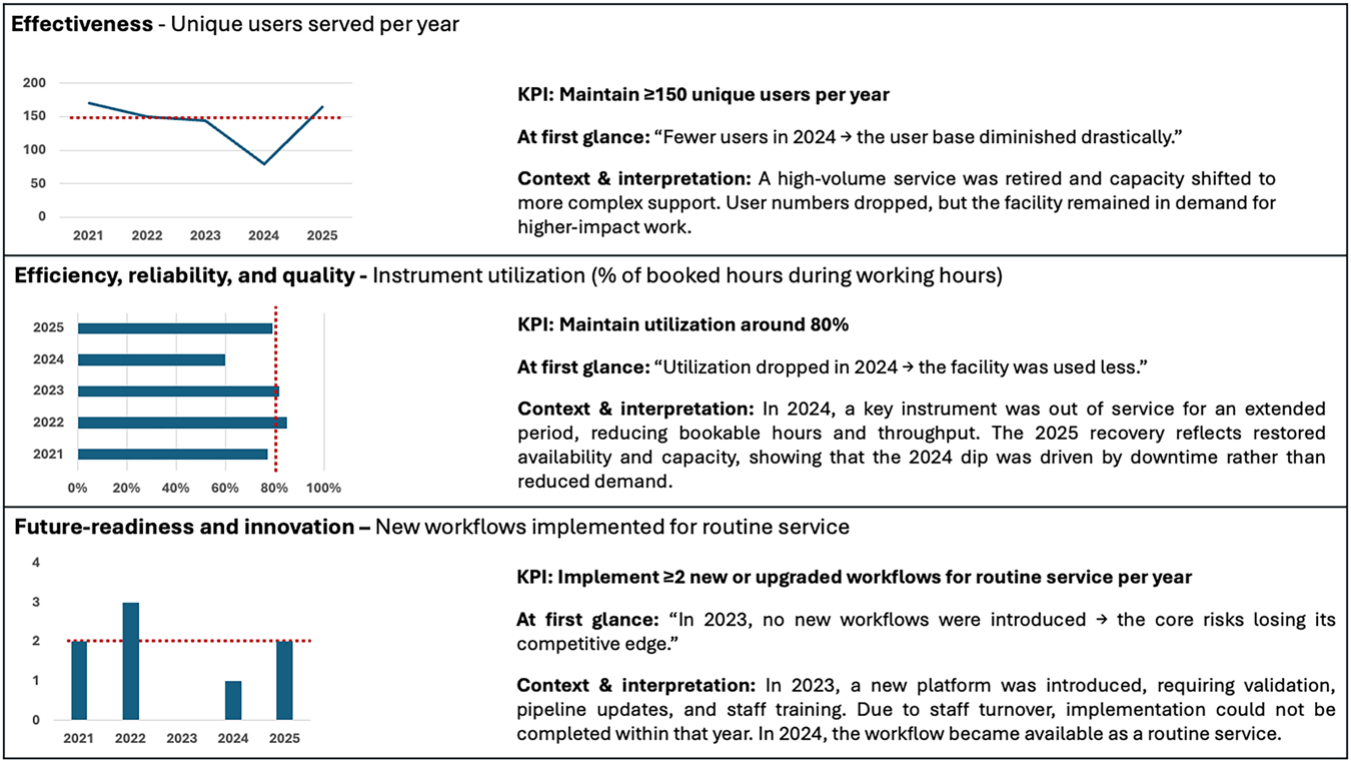

## Supplementary information

Peer Review File

## References

[CR1] Clissold KA, Itano MS (2025) How do you measure the success and impact of a core facility? Biotechniques 77:263–270 40931915 10.1080/07366205.2025.2555118 PMC12639534

[CR2] Gregory CW (2020) Building a quality management system in a core facility: a genomics core case study. J Biomol Tech 31:57–65 32655325 10.7171/jbt.20-3102-004 PMC7266070

[CR3] Kivinen K, van Luenen H, Alcalay M, Bock C, Dodzian J, Hoskova K, Hoyle D, Hradil O, Christensen SK, Korn B, Kosteas T, Morales M, Skowronek K, Theodorou V, Van Minnebruggen G, Salamero J, Premvardhan L (2022) Acknowledging and citing core facilities: key contributions to data lifecycle should be recognised in the scientific literature. EMBO Rep 23:e55734 35997112 10.15252/embr.202255734 PMC9442286

[CR4] Kos-Braun IC, Gerlach B, Pitzer C (2022) Assessing and improving research quality in core facilities. J Biomol Tech 33:3fc1f5fe.97057772 35837001 10.7171/3fc1f5fe.97057772 PMC9258915

[CR5] Meder D, Morales M, Pepperkok R, Schlapbach R, Tiran A, Van Minnebruggen G (2016) Institutional core facilities: prerequisite for breakthroughs in the life sciences: core facilities play an increasingly important role in biomedical research by providing scientists access to sophisticated technology and expertise. EMBO Rep 17:1088–1093 27412771 10.15252/embr.201642857 PMC4967956

[CR6] Moore J, Roederer M (2009) The flow cytometry shared resource laboratory: best practices to assure a high-quality, cost-effective partnership with biomedical research laboratories. Cytometry A 75:643–649 19582865 10.1002/cyto.a.20742

[CR7] O’Toole PJ, Marrison JL (2024) A perspective into full cost recovery within a core facility/shared resource lab. J Microsc 294:372–379 37973413 10.1111/jmi.13246

[CR8] Turpen PB, Hockberger PE, Meyn SM, Nicklin C, Tabarini D, Auger JA (2016) Metrics for success: strategies for enabling core facility performance and assessing outcomes. J Biomol Tech 27:25–39 26848284 10.7171/jbt.16-2701-001 PMC4736753

