# Supplementary material for: Counting more than beans: A different take on key performance indicators for core facilities
Source: EMBO Rep. 2026 Apr 8;27(9):2156–9. doi: 10.1038/s44319-026-00762-z (PMC13172349; doi:10.1038/s44319-026-00762-z)
Supplement: Supplementary file 1 — Peer Review File [file 44319_2026_762_MOESM1_ESM.pdf]

EMBO reports

Counting more than beans: A different take on key performance indicators for core facilities

Farzaneh Ghazavi, Alexander Botzki, Stefaan Derveaux, Christine Durinx, Gert Van Isterdael, Geert Van Minnebruggen, and Saskia Lippens

Corresponding author(s): Saskia Lippens (Saskia.Lippens@vib.be)

Review Timeline:

Submission Date:	11th Mar 26
Accepted:	24th Mar 26

Editor: Holger Breithaupt

Transaction Report: This article was editorially reviewed.
